# Supplementary material for: Comprehensive Organ-Specific Profiling of Douglas Fir (Pseudotsuga menziesii) Proteome
Source: Biomolecules. 2023 Sep 16;13(9):1400. doi: 10.3390/biom13091400 (PMC10526743; doi:10.3390/biom13091400)

Supporting Information Figure S1.

Scaled Principal Component Analysis of all the biological replicates of proteomes of Douglas-fir organs with (**a**) individual plot of the proteomic quantitative analysis and (**b**) the correlation circle plot associated (proteins are represented by dots). Values for x and y axes are those of PC1 and PC2, respectively.

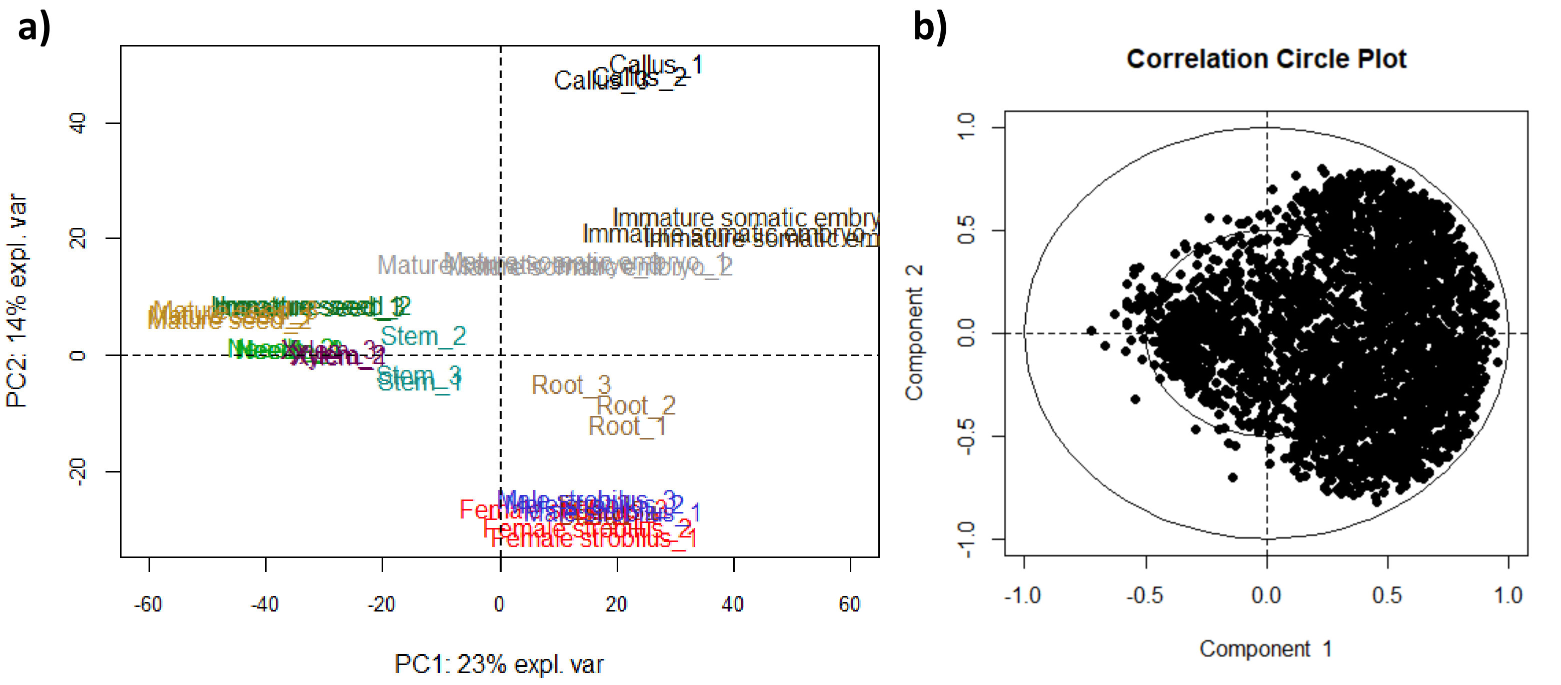

Supporting Information Figure S2.

Scaled Principal Component Analysis of the GO abundance of the molecular function of the 12 proteomes of Douglas-fir with (a) the individual plot and (b) the correlation circle plot associated with GO terms. Values for x and y axes are those of PC1 and PC2, respectively.

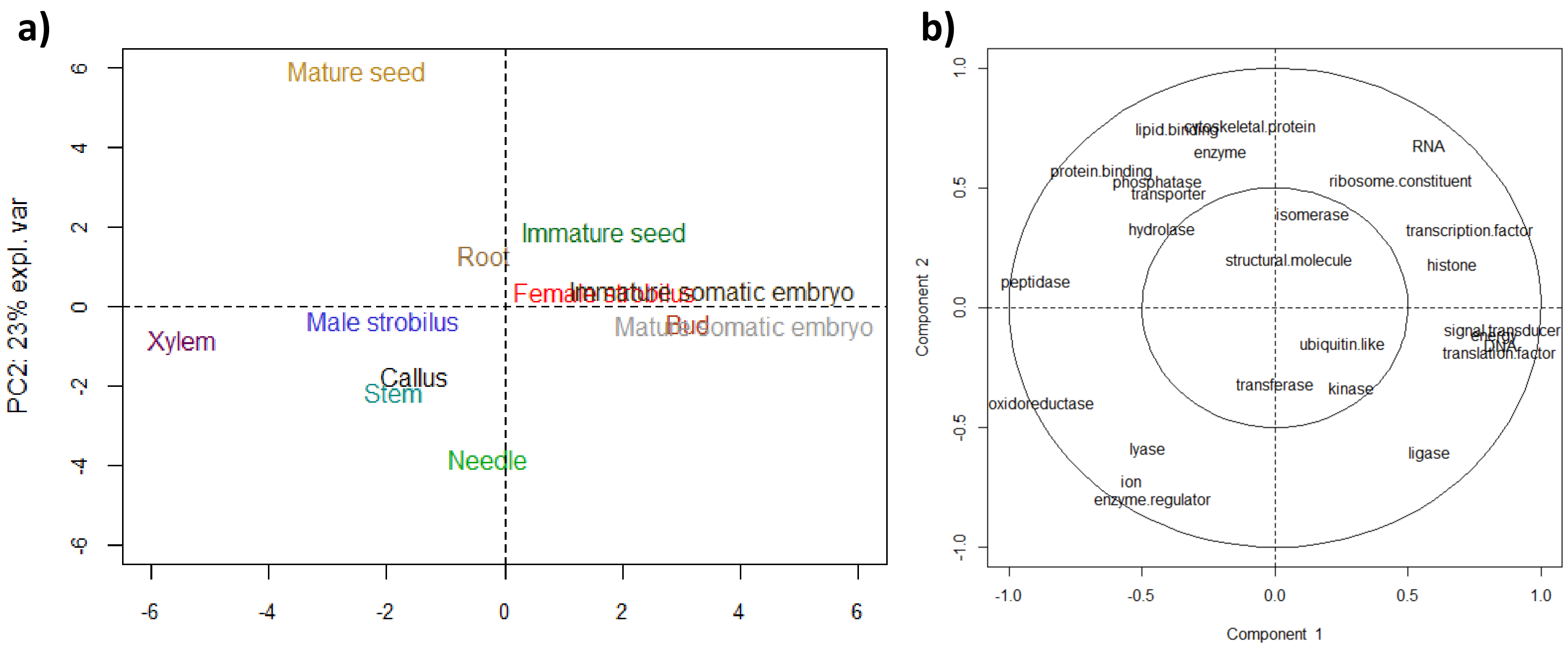

Supplement: Supplementary file 1 [file biomolecules-13-01400-s001.zip › Teyssier et al Figures_Supp.pdf]
